# Fabrication and Electrochemical Performance of PVA/CNT/PANI Flexible Films as Electrodes for Supercapacitors

**DOI:** 10.1186/s11671-020-03379-w

**Published:** 2020-07-22

**Authors:** Jianwei Ben, Zhiyuan Song, Xinke Liu, Wei Lü, Xiaohua Li

**Affiliations:** 1 College of Materials Science and Engineering, College of Electronics and Information Engineering, Guangdong Research Center for Interfacial Engineering of Functional Materials, Shenzhen University – Hanshan Normal University postdoctoral workstation, Shenzhen University, Shenzhen, 518060 China; 2 College of Physics and Optoelectronic Engineering, Shenzhen University, Shenzhen, 518060 China; 3 Key Laboratory of Advanced Structural Materials, Ministry of Education &Advanced Institute of Materials Science, Changchun University of Technology, Changchun, 130012 China

**Keywords:** Carbon nanotube, Polyaniline, Polyvinyl alcohol, Flexibility, Supercapacitor

## Abstract

**Abstract:**

The flexible and rechargeable energy storage device with excellent performance is highly desired due to the demands of portable and wearable devices. Herein, by integrating the bendability and stretchability of Polyvinyl alcohol (PVA), pseudocapacitance of Polyaniline (PANI), and the charge transport ability of carbon nanotubes (CNTs), PVA/CNT/PANI flexible film was fabricated as supercapacitor electrodes with excellent electrochemical performance and flexibility. Full-solid supercapacitor is prepared based on PVA/H_2_SO_4_ gel electrolyte and as-prepared film electrodes. The device achieves an areal capacitance of 196.5 mF cm^-2^ with high cycling stability. The flexible properties of PVA, the conductivity of CNT, and the pseudo-capacitance of PANI contribute to the superior performance. Present work develops a facile and effective way for preparing flexible electrode materials.

**Graphical Abstract:**

In present work, we fabricated PVA/CNT/PANI flexible film as supercapacitor electrodes with excellent electrochemical performance and flexibility. 
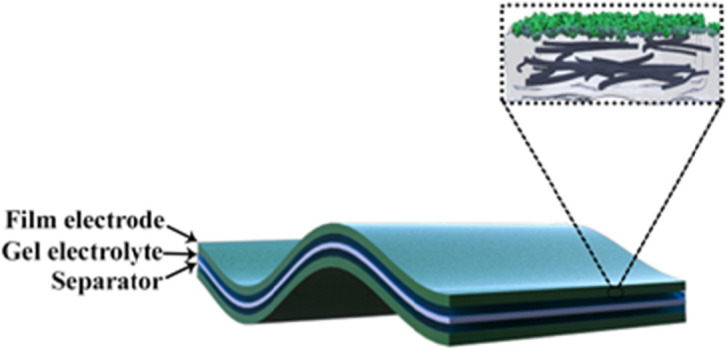

## Highlights

- A facile and economic strategy was developed to prepare PVA/CNT/PANI flexible film as supercapacitor electrodes.
- The device achieves an areal capacitance of 196.5 mF cm^−2^ with high cycling stability.
- The flexibility of PVA, the conductivity of CNT, and the pseudo-capacitance of PANI are contributed to the superior performance.

## Introduction

The portable and wearable electronic devices are receiving great attention in recent years [1, 2], which requires the miniature power supply source integrated with flexibility. The requirements of miniaturization and bendability limit the size and thickness of power supply, and thus sacrificing the durability to a great extent [3, 4]. Supercapacitor as a kind of typical energy storage device is characterized by simple structure, high power density, fast charging and discharging speed, and excellent reliability [5–8], and is promising for applications in portable and wearable electronic devices [9]. While the flexible device of supercapacitor could be realized by material design [1, 10–12], great efforts have been made to develop flexible supercapacitors in recent years for enhancing energy density. The critical point is to bear large deformation strain while maintaining the satisfied storage performance [12–14].

Carbon materials, conductive polymers, and metal oxides or their composites are most generally used electrode materials of supercapacitors [15–19]. Carbon nanotubes (CNTs) are a good choice because of their good conductivity, good charge transport, and high mechanical strength. The p electrons of carbon atoms form a large range of delocalized π bonds, and the conjugation effect is significant, so CNTs have good conductivity [20]. However, CNTs belong to the mechanism of double electric energy storage, which only relies on the electronic adsorption to provide the capacitance performance, and exhibited poor capacitor performance as electrodes, which may seriously limit their applications. In this regard, CNTs could be dispersed in polymer matrix served as an additional path for charge transfer as electrode materials [21–23].

For pseudocapacitive materials, conductive polymers have attracted great attention due to their large theoretical capacitance, better capacity retention, low toxicity, and eco-friend [24–26]. The excellent conductivity, electrochemical performance, and stability make polyaniline (PANI) is considered as an ideal choice for electrode materials. Unfortunately, due to PANI’s poor mechanical properties, it is hard to obtain high conductivity and stretchability at the same time [27, 28]. Compared with PANI, polyvinyl alcohol (PVA) based hydrogel is softer and has been applied as a solid electrolyte. Furthermore, the PVA hydrogel exhibits appreciable mechanical strength [29–32]. Hu et al. improved mechanical properties through combing PVA and PANI as electrode materials of the stretchable supercapacitor [33]. Faraji et al. used polyvinyl chloride, CNTs, and PANI to construct composite films as electrode materials of flexible supercapacitor [34]. Li et al. prepared a flexible solid-state supercapacitor based on graphene/polyaniline paper electrodes and showed good electrochemical performance [35]. Yang et al. used PEDOT and PANI conductive polymer as electrode materials to prepare flexible supercapacitor with excellent electrochemical performance [36].

In present work, PVA/CNT/PANI flexible film was prepared as supercapacitor electrodes. CNTs are used as a charge transfer pathway to enhance the conductivity of polymer and double-layer capacitance. PANI polymer provides pseudocapacitance, and the PVA matrix provides bendable and stretchable ability. Flexible solid symmetric supercapacitor was assembled by PVA/CNT/PANI film as active electrodes, and achieves capacitance of 196.5 mF cm^−2^. There is still 71.4% capacitance retention rate after 5000 cycles, which exhibited excellent cycling stability. Present work provided high universality to develop high strain polymeric materials with excellent electrochemical properties.

## Experimental Section

### Materials

CNT was supplied from Aladdin Reagent Co., Ltd. PVA, ammonium persulfate (APS), aniline (ANI), ethyl alcohol, hydrochloric acid, sulfuric acid (98%), and nitric acid (96%) were purchased from Aladdin.

### Preparation of Flexible PVA/CNT/PANI Film

The dispersion of CNTs in the solution is very poor, and there will be an obvious agglomeration phenomenon. Using strong acid oxidation and ultrasonic treatment can improve the dispersion of CNTs in solution. Typically, 500 mg CNT was added into the 40-ml mixed solution of HNO_3_ and H_2_SO_4_ in volume ratio of 1:3 under sonication. Then, it was heated to 90 °C for 120 min. The acidulated CNT was washed with deionized water and dried at 80 °C for 6 h. PVA/CNT film was fabricated using the following sententious process.

**Fig. 1:**
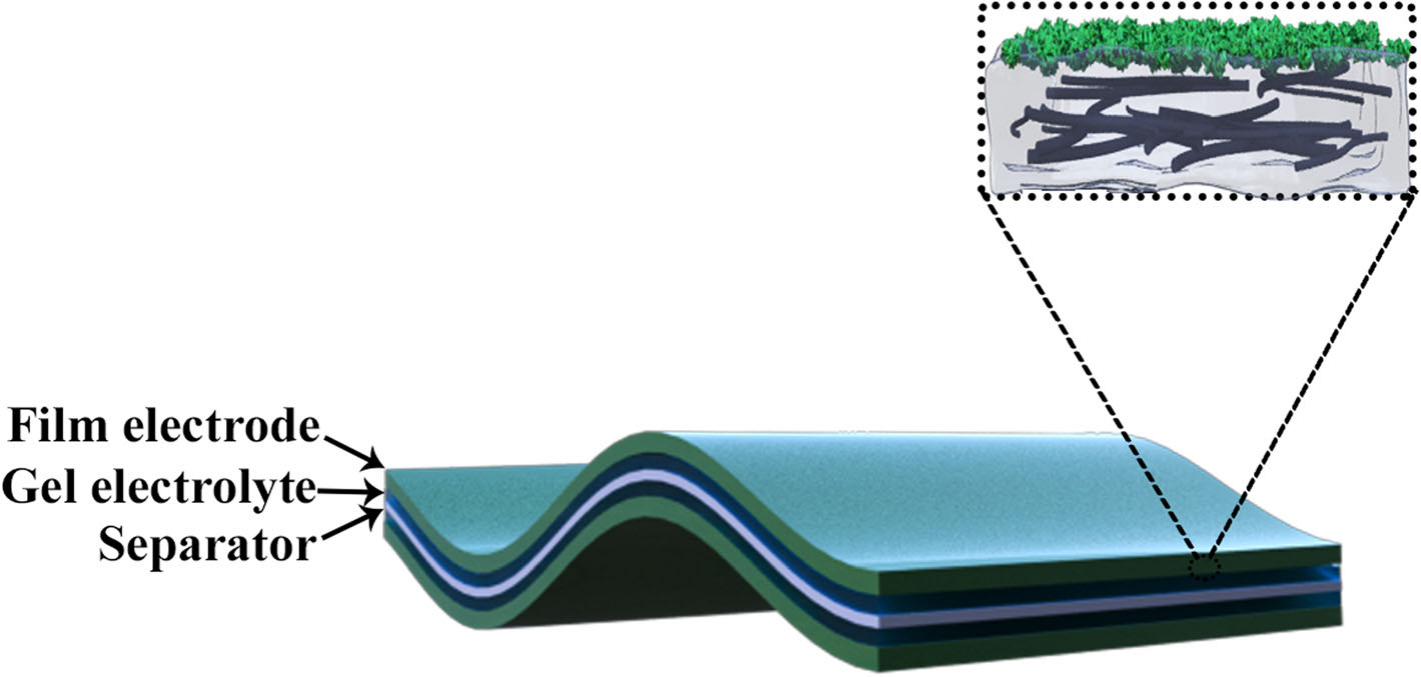
Schematic illustration of symmetric supercapacitor based on PVA/CNT/PANI electrodes and gel electrolyte

As shown in Fig. 1, composite films were prepared by in situ polymerization of PANI on the surface of PVA/CNT films. Firstly, a 10-ml aqueous solution including 500 mg PVA was prepared, and 50 mg CNTs were added successively. After heating to 95 °C for 30 min, the mixture was poured into a petri dish, and left overnight. For PVA/CNT/PANI film, the PVA/CNT film was immersed in 10-mL aniline (0.5 M) for 10 min, followed by adding 10 mL ammonium persulfate (APS) solution (0.5 M). The PVA/CNM/PANI films were obtained after different reacting times of 6, 9, and 12 h.

### Preparation of Flexible Solid-state Supercapacitors

The PVA/H_2_SO_4_ gel was used as electrolyte. A 10-ml aqueous solution including 1 g PVA was prepared under vigorous stirring, which is followed by addition of 0.56 ml of H_2_SO_4_. The acquired PVA/H_2_SO_4_ gel is agreed to cool. The as-prepared PVA/CNT/PANI films were applied on both sides of PVA/H_2_SO_4_ gel to complete assembly of a typical supercapacitor with carbon cloth as the current collector.

### Characterization

The acquired samples and devices are investigated by field emission scanning electron microscopy (SEM, 7610, JEOL, Japan), Raman spectra and Fourier transform infrared (FTIR) spectra (Thermo Electron Scientific Instruments, USA). The electrochemical impedance spectroscopy (EIS), galvanostatic charge-discharge (GCD), and cyclic voltammetry (CV) were characterized by CHI660E electrochemical workstation (Shanghai Chen-hua instrument co. LTD).

## Results and Discussion

**Fig. 2:**
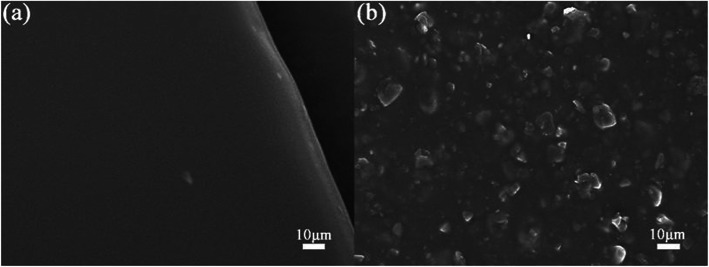
SEM images of pure PVA film (**a**) and PVA/CNT film (**b**)

As shown in Fig. 1, the conductive scaffold was formed by the uniform dispersion of CNTs which was used as a charge transfer pathway to enhance the conductivity of polymer and double-layer capacitance. PANI provides pseudocapacitance, and the PVA matrix provides bendability and stretchability. Figure 2a demonstrates a top-view SEM image of pure PVA film. It could confirm the existence of a smooth surface due to the polymer feature [37]. To assure better dispersion of CNTs in PVA, the CNTs are treated by acid before using. As shown in Fig. 2b, the rougher surface of PVA/CNT film compared with that of pure PVA film could be confirmed, indicating the blending of CTNs.

**Fig. 3:**
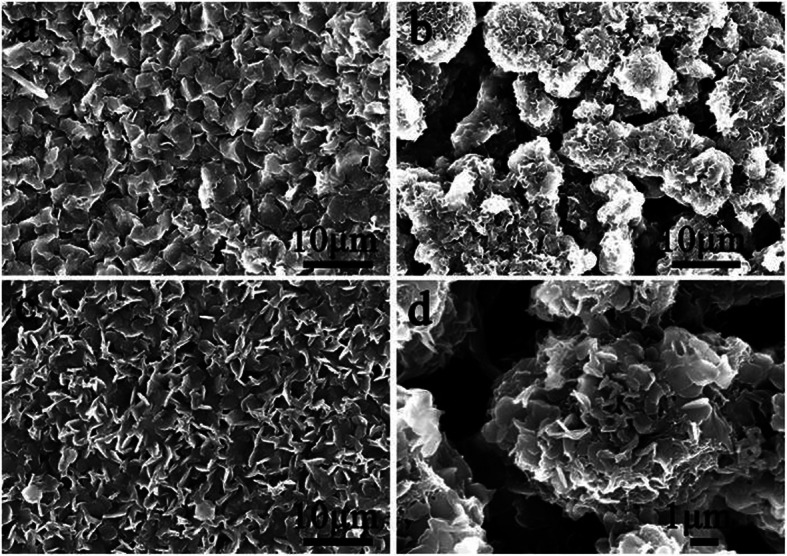
SEM diagram of composite films at different polymerization times (**a**) 6 h, (**b**) 9 h, (**c**) 12 h, and (**d**) an enlarged view of 9 h

After covering PANI on PVA/CNT film, different surface morphology could be observed with complicated microstructure as shown in Fig. 3. With the increasing polymerization time, the morphologies of films changed gradually. In Fig. 3a and c, it can be seen that PANI with lamellate structures is evenly distributed on the surface of the composite film, indicating the 6- and 12-h samples have similar morphologies. The density of lamellate structures for 12-h sample is higher than that of 6-h sample, which suggests the amount difference of PANI in the composite films with different polymerization time, and should induce different electrochemical behaviors. However, it should be noticed that the 9-h sample exhibits very special morphology compared with that of 6- and 12-h samples, as shown in Fig. 3b and d, a flower-like structure composed of small flakes could be observed. The formation of special morphology could be due to intermolecular force-induced self-assembling at a certain amount of PANI during polymerization, which would be destroyed with an excessive supply of ANI molecules. It could be observed from Fig. 3d that the cracks among flowers provides enough space for wetting between active materials and electrolyte, and thus could improve the reaction and storage ability of electrons [38–40].

**Fig. 4:**
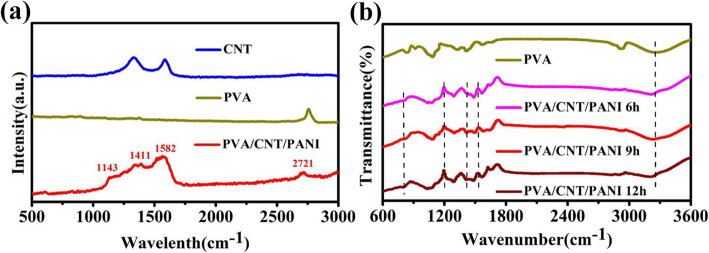
**a** Raman spectra of CNT, pure PVA, and PVA/CNT/PANI film. **b** FTIR spectra of PVA and PVA/CNT/PANI films

Figure 4a is the Raman spectra of samples. For CNTs, two bands at 1346 and 1587 cm^−1^ correspond to disordered carbon and ordered graphitic sp^2^ carbon, respectively. For PVA, the obvious peak is much less, and the peak around 2847 cm^−1^ is attributed to -CH_2_ [41]. For PVA/CNT/PANI film, the peak at 1143 cm^−1^ is originated from vibration of the C-H bond, and two peaks at 1411 and 1582 cm^−1^ could be contributed by C-C [42]. The peak at 2721 cm^−1^ deviated from the peak position of PVA, which may be due to the overlap of PANI on PVA. Figure 4b is the FTIR spectra of PVA and PVA/CNT/PANI film. The three PVA/CNT/PANI films show similar feature peaks. The characteristic absorption peaks at 1202 cm^−1^ results from C-N bond. A series of peaks in the range of 1430-1530 cm^−1^ is due to C=C stretching vibration [43]. A broad peak corresponds to the stretching vibration of -OH group at 3255 cm^−1^. These results indicate the successful preparation of desired composite films. The different polymerization time induces different film morphology and loading amount of PANI, so the electrochemical performance of devices based on these films are further investigated.

**Fig. 5:**
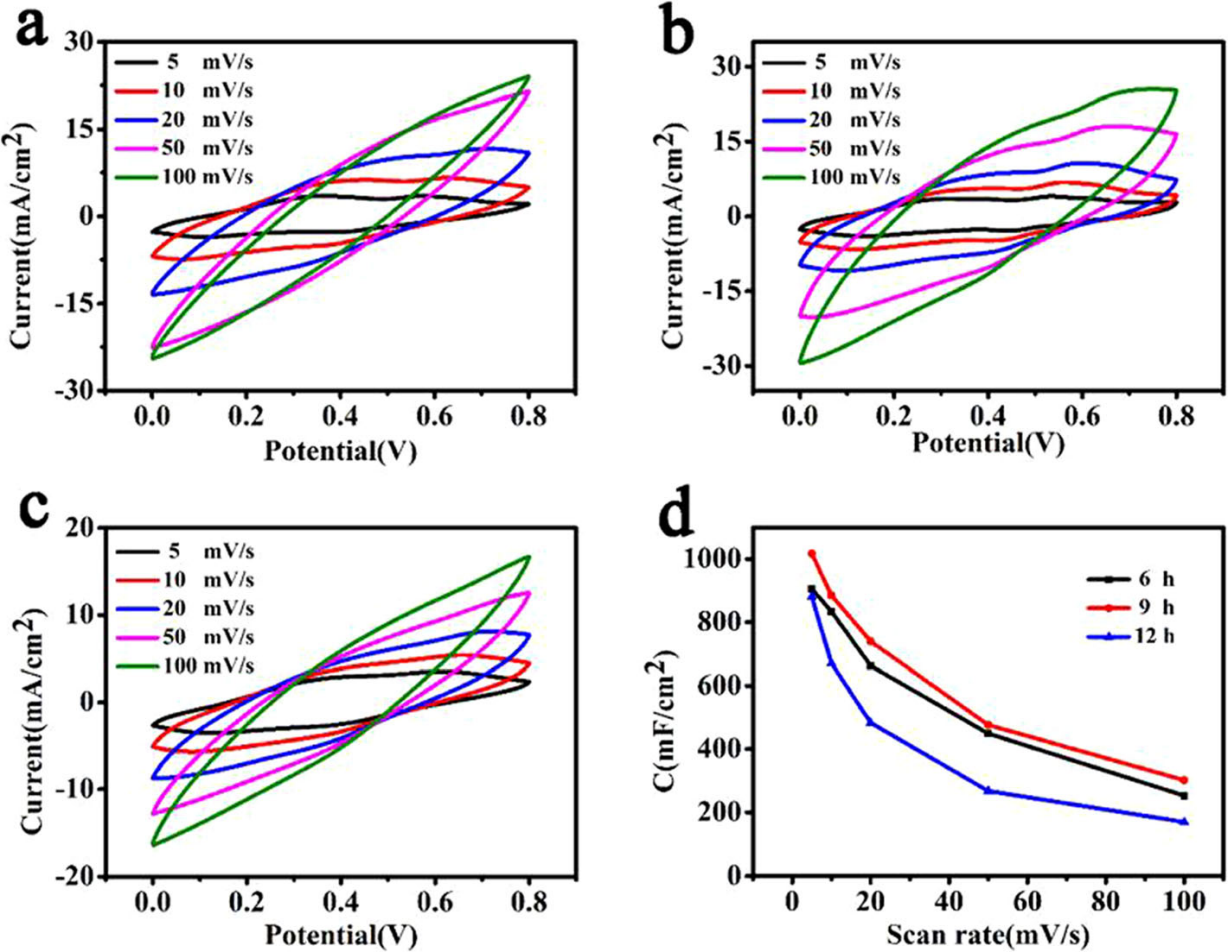
The CV curves of the PVA/CNT/PANI film (**a**) 6 h, (**b**) 9 h, (**c**) 12 h; (**d**) specific capacitances of PVA/CNT/PANI film with increasing scan rate

The electrochemical properties of the PVA/CNT/PANI samples were evaluated by the three-electrode test. The composite films prepared by cutting in situ polymerization sample were used as a working electrode, and the CV curves were measured. Figure 5 shows the test results of different samples, the shapes of corresponding curves are consistent very well with that of CV curves of polymer as supercapacitor electrode materials [44, 45]. The largest area of CV curves is achieved by a 9-h sample as shown in Fig. 5b. It is found that the generation of redox peaks belong to the energy storage mechanism of pseudocapacitor, which further proves the existence of PANI. The area-specific capacitance of samples as a function of scanning rate is shown in Fig. 5d. For the 9-h sample, the area-specific capacitance of PVA/CNT/PANI composite film electrode at a scanning rate of 5 mV s^−1^ is 1016.8mF cm^−2^, and that of 6 h and 12 h are 906 mF cm^−2^ and 881.3 mF cm^−2^, respectively. The results indicate that the 9-h sample shows the best performance which is consistent with the observation of SEM that the cracks among flowers provide enough space for wetting between active materials and electrolyte, and thus could improve the reaction and storage ability of electrons.

**Fig. 6:**
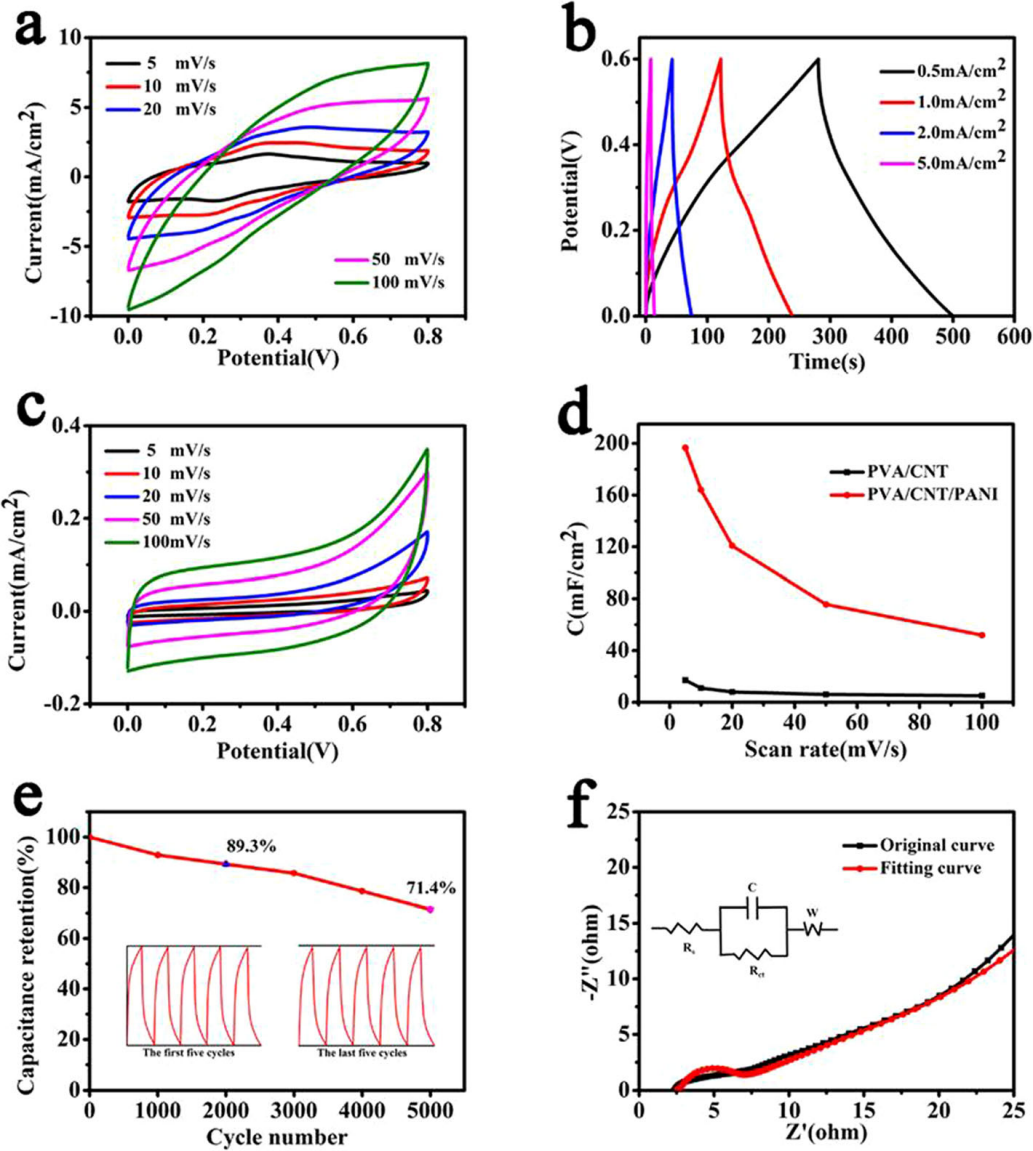
**a** The CV curves of the 9 h PVA/CNT/PANI-based supercapacitor and PVA/CNT based supercapacitor (**c**). **b** Curves of GCD with different current densities of the 9 h PVA/CNT/PANI. **d** Specific capacitances of PVA/CNT, PVA/CNT/PANI based supercapacitor with increasing scan rate. **e** Cyclic stability measurement and the insets are cycling curves of the first five cycles and the last five cycles, respectively. **f** Nyquist plots of the device

The working status of PVA/CNT/PANI films as electrodes in a flexible supercapacitor is further investigated and a full-solid supercapacitor is fabricated as described in the experimental section using a 9-h PVA/CNT/PANI film. The device has a typical sandwich structure. As seen in Fig. 6a, symmetrical shapes of CV curves are kept well with the increasing scan rate, indicating the ideal capacitive behavior of PVA/CNT/PANI film [46–48]. The supercapacitor obtains the highest areal capacitance (196.5 mF cm^−2^) at a scanning speed of 5 mV s^−1^. For the flexible PVA/CNT/PANI symmetrical supercapacitor, the capacitance performance is also tested by GCD in Fig. 6b, showing linear and symmetrical triangles, indicating excellent capacitive performance [49]. To confirm the effect of PANI on enhancing energy storage ability, CV curves of PVA/CNT-based supercapacitor are also measured as shown in Fig. 6c. Although the symmetrical shapes are remained, the specific capacitance of PVA/CNT electrodes is much smaller compared with that of PVA/CNT/PANI film electrode as shown in Fig. 6d. Cyclic stability of device is measured by constant current charge/discharge. After 2000 cycles, the PVA/CNT/PANI-based supercapacitor achieves 89.3% capacitance retention, and 71.4% capacitance retention after 5000 cycles as shown in Fig. 6e. With the increase of working time, the structure of electrode materials and solid electrolyte would be affected. The electrode materials would gradually dissolve in electrolyte during the cycling process, resulting in the gradual attenuation of capacitance. The EIS spectra of the flexible solid-state supercapacitor is measured in the frequency range from 0.01 Hz to 100 KHz [50–53] as shown in Fig. 6f, and the inset is an equivalent circuit. The small values of Rs and Rct indicate small electrode resistance and a high charge transfer rate [54]. Table 1 shows the comparison of present work with other reports, it could be confirmed that the present PVA/CNT/PANI film as flexible electrodes of supercapacitor achieved excellent performance [55–59].

**Table 1:** Summary of the capacitive performance of the supercapacitors based on similar materials

Sample	Fabrication method	C_**a**_(mF cm^**−2**^)	T	C (%)	Ref
PVA/CNT/PANI film	In situ polymerization	196.5	5000	71.4	Present work
RGO/PPy	In situ reduction polymerization	175	5000	93	[55]
PANI/G paper	Electrochemical polymerization	123			[35]
CNT/PANI film	Electrodeposition	184.6	500	95	[56]
PVA/PANI hydrogel	Hydrogel mixing	11.3			[33]
CNT/G film	Suction filtration	11	4500	92	[57]
PEDOT/PANI	Acid treatment polymerization	118	5000	82.5	[36]
PVA/CNT@Ni(HCO_3_)_2_	Hydrothermal synthesis	143.6	2000	85.5	[58]
PPy/G/Ni-foam	In situ polymerization	165	1500	94.5	[59]

Abbreviations: *C*_*a*_ specifific capacitance; *C* retention rate of cycle life test; *CNT* carbon nanotube; *G* graphene; *T* cycles of the cycle life test

## Conclusions

In summary, PVA/CNT/PANI film was prepared by a facile method. Due to the synergistic effect of PVA, CNT, and PANI, the obtained films have good flexibility, bendability, and electrochemical properties. The area capacitance reached 196.5mF cm^−2^ and after 5000 cycles, the capacitance retention rate reached 71.4%, showing good cyclic stability. The flexible properties of PVA, the conductivity of CNT, and the pseudo-capacitance of PANI contribute to the superior performance. Present work provided a simple but efficient method for the preparation of flexible electrode materials.

## Data Availability

The datasets used or analyzed during the current study are available from the corresponding author on reasonable request.

## Abbreviations

PANI: Polyaniline
PVA: Polyvinyl alcohol
CNTs: Carbon nanotubes
CV: Cyclic voltammetry
SEM: Scanning electron microscopy
FTIR: Fourier transform infrared spectrum
EIS: Electrochemical impedance spectroscopy
GCD: Galvanostatic charge-discharge spectrum
